# Genomic Characterization of an O-Antigen-Deficient, Hydrogen Sulfide-Negative *Salmonella enterica* Serovar Senftenberg Isolated from Cooked Mussels

**DOI:** 10.3390/microorganisms14061284

**Published:** 2026-06-06

**Authors:** Alexandre Lamas, Antonio Lozano-León, Alejandro Garrido-Maestu, Narjol Gonzalez-Escalona

**Affiliations:** 1 Food Hygiene, Inspection and Control Laboratory (Lhica), Department of Analytical Chemistry, Nutrition, and Bromatology, Veterinary School, Campus Terra, Universidade de Santiago de Compostela (USC), 27002 Lugo, Spain; alexandre.lamas@usc.es; 2 Health & Nutrition Laboratory Manager, SGS Seafood Laboratory, 36939 Bueu, Spain; antoniobarbaro.lozanoleon@sgs.com; 3 Laboratory of Microbiology and Technology of Marine Products (MicroTEC), Institute of Marine Research (IIM-CSIC), 36208 Vigo, Spain; 4 Genomics Development and Applications Branch, Division of Food Safety Genomics, Office of Applied Microbiology and Technology (OAMT), Office of Laboratory Operations and Applied Science (OLOAS), Human Foods Program, Food and Drug Administration, College Park, MD 20740, USA

**Keywords:** *Salmonella enterica*, atypical strains, somatic antigens, H_2_S-negative, whole-genome sequencing, hybrid assembly, mussels, food safety, One Health

## Abstract

Atypical *Salmonella enterica* strains that evade conventional detection pose significant challenges to food safety surveillance. A hydrogen sulfide (H_2_S)-negative and serologically untypable *S. enterica* strain (SF1060) was detected by qPCR from cooked farmed mussels in Galicia, Spain, and characterized using phenotypic and genomic approaches. Despite typical biochemical profiles, SF1060 failed to produce black colonies on Xylose Lysine Deoxycholate (XLD) agar and lacked detectable somatic antigens by conventional serotyping. Hybrid genome assembly using nanopore and illumina sequencing yielded a closed chromosome and five plasmids. In silico analyses identified the strain as *S.* Senftenberg ST14. Comparative genomics revealed a chromosomal inversion at the *rfb* operon (encoding enzymes needed to synthesize deoxysugars and O antigens) mediated by *IS5*-family transposase *ISEc68*, which truncated the *rfbD* gene and separated the remaining *rfb* genes at *rfb*D, disrupting O-antigen biosynthesis, explaining the inconclusive phenotypic serotyping results. The *phs* operon responsible for H_2_S production lacked premature stop codons, suggesting the H_2_S-negative phenotype may result from an alternative mechanism. This study demonstrates how whole-genome sequencing resolves identification of atypical strains that fail culture-based detection and emphasizes the critical need for molecular surveillance methods in seafood safety programs, particularly in regions where atypical *S. enterica* variants may be endemic.

## 1. Introduction

*Salmonella enterica* is one of the most common pathogens responsible for foodborne illnesses worldwide [1]. More than ~80,000 cases are annually reported in Europe [2] and more than 1 million in the US [3]. These figures highlight the importance of detecting and accurately characterizing *Salmonella enterica* for food safety. Its detection in foodstuffs is based on culture-based methods such as ISO 6579 or BAM Chapter [4,5]. Even though these methods are highly reliable, their performance is reduced when dealing with atypical strains that may not grow on selective media with the expected appearance. The implementation of molecular methods, such as real-time PCR (qPCR), which are not affected by the phenotype, is a way to overcome this limitation of classical methods.

Mussel aquaculture is a key industry in Spain, which is ranked among the top five producers worldwide, and more specifically in Galicia. This region is located in the northwest of the country, and it is responsible for more than 90% of the production of this product [6]. The marine environment presents unique challenges for *S. enterica* surveillance, as the pathogen can persist in seawater, biofilms, and filter-feeding shellfish, creating complex transmission networks that link environmental reservoirs, wildlife, and human consumers. These figures highlight the importance of assuring the safety of the product to safeguard the consumers and avoid the potential economic impact due to potential foodborne diseases.

The presence of atypical *S. enterica* strains has already been reported in this area [7], and even strains bearing genes providing resistance to last-resort antibiotics like colistin [8,9]. This fact indicates that the implementation of phenotype-independent techniques is needed in order to better control this pathogen. In this sense, methodologies based on the techniques previously mentioned, PCR/qPCR, are of high interest, but the advancement of novel molecular techniques, such as whole-genome sequencing (WGS), provides a comprehensive tool for characterizing atypical strains, offering insights into their genetic basis and improving surveillance efforts [10].

During routine testing for the presence of *S. enterica* in mussels from Galicia, Spain, a particular strain was detected that failed to produce the typical black colonies on Xylose Lysine Deoxycholate (XLD) agar, despite being identified as *S. enterica* by qPCR. Subsequent analysis at a reference laboratory revealed that this strain was untypable by conventional serotyping methods. These unusual phenotypic and serological characteristics prompted a detailed genomic investigation to elucidate the genetic basis underlying the absence of hydrogen sulfide (H_2_S) production and the untypable serotype. Here, we describe the genomic features of this atypical strain and discuss the possible molecular mechanisms responsible for its distinctive phenotype. The objectives of this study were to: (i) characterize the genomic features of an atypical H_2_S-negative *S. enterica* strain from cooked mussels; (ii) identify the genetic mechanisms responsible for loss of somatic antigen expression and H_2_S production; and (iii) assess the phylogenetic relationship of this strain to other *S.* Senftenberg strains from the region.

## 2. Materials and Methods

### 2.1. Sample Collection and Bacterial Isolation

Samples were collected as part of routine microbiological monitoring of mussel production facilities in Galicia, Spain, during July 2019–July 2020. A total of 1665 samples were screened during this surveillance period. *S. enterica* strain SF1060 was isolated from routine microbiological controls of mussels. Briefly, 25 g of mussel was mixed with 225 mL of 2% Buffered Peptone Water (BPW, BioMérieux, S.A., Marcy l’Etoile, France) and homogenized for 90 s in a stomacher; then the mixture was incubated at 37 °C for 18 ± 2 h. The DNA from the enriched samples was extracted with the PrepSEQ™ Rapid Spin Sample Preparation Kit (Applied Biosystems, Foster City, CA, USA) and then screened for *S. enterica* using the MicroSEQ™ *Salmonella enterica* detection kit (Applied Biosystems, Foster City, CA, USA) following the protocol provided by the manufacturer in a 7500 Fast Real-Time PCR System Thermal Cycler (Applied Biosystems, Foster City, CA, USA). Samples returning a positive qPCR result were confirmed by streaking the enriched sample on XLD (BioMérieux, S.A., Marcy l’Etoile, France) and Brilliance™ *Salmonella* agar (OXOID, Hampshire, UK). The plates were incubated at 37 °C overnight. Typical *S. enterica* colonies were re-isolated onto Tryptic Soy Agar (TSA, Merck KGaA, Darmstadt, Germany) and incubated at 37 °C overnight. For *S. enterica* confirmation, the biochemical profile was attained with the API20E (BioMérieux, S.A., Marcy l’Etoile, France), and the agglutination test was performed with the *Salmonella* test kit (Oxoid Diagnostic Reagents, Oxoid, UK). *S. enterica* serotyping was performed using the Kauffman–White typing scheme by slide agglutination for the detection of somatic (O) and flagellar (H) antigens with standard antisera in an external accredited laboratory. *S. enterica* strain SF1060 was conserved in cryovials at −20 °C until use.

### 2.2. DNA Isolation and Quantification

For DNA isolation, *S. enterica* strain SF1060 was streaked in a Brain–Heart Infusion (BHI) agar plate (Merck Millipore, Darmstadt, Germany) and incubated 24 h at 37 °C. A single colony was resuspended in 10 mL of BHI broth and incubated at 37 °C for 18 h at 130 rpm. Finally, 2 mL was transferred to a microtube and centrifuged at 16,000× *g* for 2 min. The supernatant was discarded, and the pellet was used for DNA extraction with the PureLink™ Genomic DNA Mini Kit (Invitrogen, Carlsbad, CA, USA), following the protocol described for Gram-negative bacteria. Qubit™ dsDNA Quantitation, Broad Range in combination with Qubit™ 4 (ThermoFisher Scientific, Waltham, MA, USA) was used for DNA quantification.

### 2.3. Whole-Genome Sequencing

Long-read sequencing was performed using Nanopore sequencing using the MinION™ Mk1C device [Oxford Nanopore Technologies (ONT), Oxford, UK]. Briefly, 400 ng of pure DNA extracted from *S. enterica* strain SF1060 was used to prepare the DNA library using the Rapid Barcoding Sequencing kit (SQK-RBK004, Oxford Nanopore Technologies (ONT)). The sample was run in a FLO-MIN106 (R9.4.1) flow cell (FAV41850), according to the manufacturer’s instructions, for 6 h (Oxford Nanopore Technologies). The Dorado basecaller v7.1.4 was used for basecalling the run output using the high-accuracy basecalling model (HAC, basecall_model—dna_r10.4.1_e8.2_260bps_sup@v3.5.2). The reads < 4000 bp and quality scores of <9 were discarded for downstream analysis using Filtlong v0.2.1 (https://github.com/rrwick/Filtlong, accessed on 1 January 2026) with default parameters. The circular closed genome for the SF1060 strain was obtained by de novo assembly using the Flye program v2.9.2 [11], using the parameters for HAC: --nano-hq -i 4. The short-read sequencing was performed by Biomarker Technologies (BMKGENE GmbH Münster, Germany). The DNA library was prepared with VAHTS Universal DNA Library Prep Kit for Illumina V4 (Vazyme Biotech Co., Ltd., Nanjing, China) and sequenced in an Illumina NovaSeq X (Illumina, San Diego, CA, USA) with the NovaSeq X Series Reagent Kit using 2 × 150 bp paired-end chemistry according to the manufacturer’s instructions, at a 100× coverage. Reads were trimmed with Trimmomatic v0.36 [12] using default parameters.

### 2.4. Genome Assembly and Annotation

The complete circular closed polished genome for the SF1060 strain was obtained by a de novo hybrid assembly using both nanopore and illumina data for that strain with Unicycler v0.5.0 [13]. Unicycler assembled the chromosome as circular closed and oriented the chromosome to start at the *dnaA* gene. The annotation of the SF1060 genome was performed using Prokka v1.14.6 [14].

### 2.5. Bioinformatic Analyses

#### 2.5.1. In Silico Serotyping, Antimicrobial Resistance, and Virulence

MLST and serotyping. *S. enterica* MLST was determined using the Enterobase website (https://enterobase.warwick.ac.uk/species/index/senterica, accessed on 20 July 2025) and the serotype using Seqsero2 (v1.3.2, accessed on 1 January 2026) [15]. The antimicrobial resistance (AMR) gene detection was performed using ResFinder v4.6.0 (http://genepi.food.dtu.dk/resfinder, accessed on 1 January 2026) [16]. The presence of *S. enterica* Pathogenicity Islands (SPIs) was determined with the tool SPIFinder 2.0 [17].

#### 2.5.2. Phylogenetic Analysis

The phylogenetic relationship of the strains was assessed by a whole-genome multi-locus sequence typing (wgMLST) analysis using Ridom SeqSphere+ v9.0.8. The genome of *S.* Senftenberg CFSAN002050 (NC_021818.1) (4103 CDSs) was used as reference. Genes that were repeated in more than one copy in either of the two genomes were removed from the analysis as failed genes. A task template was then created that contained both core and accessory genes for each reference strain for any future testing. Each locus (core or accessory genes) from the reference strain was assigned allele number 1. The SF1060 hybrid assembly in this study was queried against the task template. If the locus was found and was different from the reference genome or any other queried genome already in the database, a new number was assigned to that locus and so on. After eliminating any loci that were missing or found having indels (shown as failed calls in the software) from the genome of any strain used in our analyses, we performed the wgMLST analysis. These remaining loci were considered the core genome shared by the analyzed strains. We used Nei’s DNA distance method to calculate the matrix of genetic distance, considering only the number of same/different alleles in the core genes. An initial neighbor-joining (NJ) tree was built using pairwise ignoring missing values and the genetic distances after the wgMLST analysis (Supplementary Figure S1). Because wgMLST is based on allele numbering rather than direct nucleotide comparison, this approach reduces the impact of recombination events in the dataset studied and enables rapid clustering of closely related genomes. The SNPs were extracted from the allele differences among the 25 genomes and used to produce an SNP matrix. This SNP matrix was used to build a phylogenetic tree inferred using the Maximum Likelihood method and Kimura (1980) 2-parameter model [18] of nucleotide substitutions and 1000 bootstraps. Evolutionary analyses were conducted in MEGA12.1 [19].

To place strain SF1060 within a broader phylogenetic context, a diverse set of 24 publicly available *S.* Senftenberg genomes was selected from the National Center for Biotechnology Information (NCBI) database for comparative analysis. The selection criteria were designed to create a representative dataset that included: (i) broad geographic diversity, with strains from North America, South America, and Europe; (ii) a wide temporal range, with isolation dates spanning several decades; and (iii) varied isolation sources, including clinical, food, and environmental samples. To specifically investigate the potential endemicity of the SF1060 lineage in the region, the dataset was supplemented with all available genomes of strains previously isolated from mussels in Galicia, Spain. The complete list of genomes, including their accession numbers, geographic origin, and isolation source, is provided in Supplementary Table S1.

#### 2.5.3. Comparative Genomics

**Figure 2:**
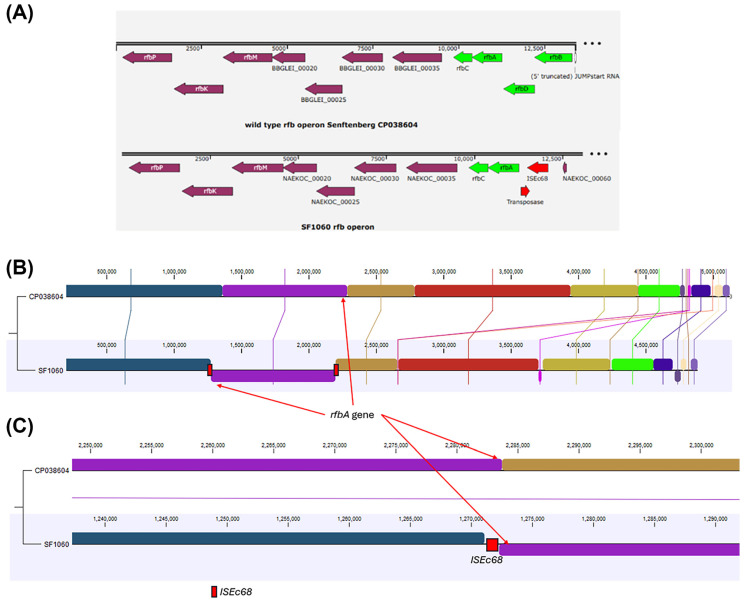
Chromosomal rearrangement at the *rfb* operon in *S. enterica* serovar Senftenberg strain SF1060 explains the loss of somatic antigen expression. Pairwise whole-genome alignment comparing SF1060 with *S.* Senftenberg strain GTA-FD-2016-MI-02533-2 (GenBank accession CP038604), which possesses an intact *rfb* operon and expresses normal O-antigens. The *rfb* gene cluster encodes the enzymes required for the biosynthesis of O-antigen deoxysugars and lipopolysaccharide assembly. (**A**) Comparison of the *rfb* operon region in CP038604 (top) and SF1060 (bottom), showing a significant structural disruption within the operon in strain SF1060. (**B**) Complete chromosome alignment showing a large-scale chromosomal inversion at the *rfb* operon in SF1060. Syntenic regions are connected by shaded bands, with inversions indicated by crossed bands. This large-scale inversion is centered on the *rfb* operon, disrupting the operon’s integrity. (**C**) Detailed view of the *rfbD* gene region showing the mechanism of disruption. In the reference strain (top), the *rfb* operon order (*rfbB*, *rfbD*, *rfbA*, *rfbC*) is intact. In SF1060 (bottom), an *IS5*-family transposase (*ISEc68* element, indicated by arrows and red colored boxes) has inserted at both ends of the inverted region, truncating the *rfbD* gene (encoding dTDP-4-dehydrorhamnose reductase) and separating the other genes of the operon from the remaining O antigen synthesis genes. This insertion of a sequence-mediated rearrangement renders the *rfb* operon non-functional, explaining the absence of detectable somatic antigens by conventional serotyping. The alignment was generated using CLC Genomics Workbench v20.

**Figure 3:**
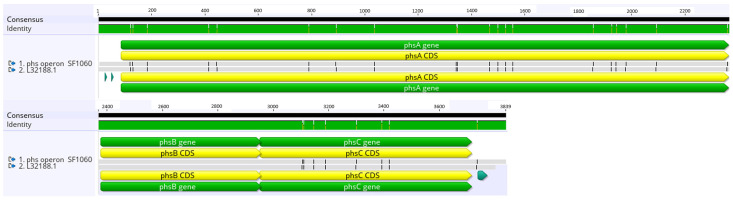
Alignment of the SF1060 *phs* operon with *S.* Typhimurium *phs* operon L32188.1. Comparative analysis of the *phs* operon in *S. enterica* serovar Senftenberg strain SF1060 and reference strain *S.* Typhimurium LT2. Nucleotide sequence alignment of the *phs* operon (thiosulfate reductase) from SF1060 compared to the reference *S.* Typhimurium LT2 *phs* operon (GenBank accession L32188.1). The *phs* operon comprises three genes (*phsA*, *phsB*, and *phsC*) responsible for the reduction of thiosulfate to hydrogen sulfide (H_2_S). Despite the H_2_S-negative phenotype of SF1060, no premature stop codons were identified in any of the *phs* genes. Missense mutations (indicated by arrows or highlighted positions) were detected in *phsA* at positions 420 (S>A), 421 (A>V), and 758 (A>V), and in *phsC* at positions 79 (L>F) and 156 (V>A). These amino acid substitutions may affect enzyme function, though their precise impact on H_2_S production remains to be determined. The alignment was generated using Geneious Prime v2025.0.3.

CLC Genomics Workbench v20 (QIAGEN, Redwood City, CA, USA) was used for creating the genome comparison of SF1060 against *S. enterica* serovar Senftenberg strain GTA-FD-2016-MI-02533-2 (CP038604) (Figure 2), using the default whole-genome alignment settings. Gene alignments shown in Figure 3 were generated in Geneious Prime v2025.0.3 (Biomatters Ltd., Auckland, New Zealand) using the default global alignment algorithm and default parameters. No non-default or manually modified alignment parameters were applied in either analysis.

## 3. Results

### 3.1. Detection and Phenotypic Characterization of Atypical S. enterica Strain

**Figure 1:**
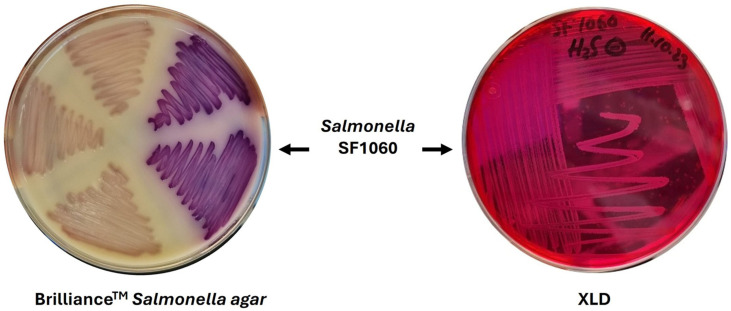
Colony morphology of *S. enterica* serovar Senftenberg strain SF1060 on selective media. (**Left**) Purple colonies on Brilliance™ *Salmonella* agar showing typical *S. enterica* morphology. (**Right**) Colonies on XLD agar lacking the characteristic black center due to the absence of H_2_S production, demonstrating the atypical phenotype that complicates culture-based detection.

During the sampling process for *S. enterica* detection from frozen cooked mussels, a qPCR positive result with a Cq value of 21 was obtained in one of the samples. This sample, however, when streaked into the selective *S. enterica* media, did not show the typical, black-centered colonies on XLD agar. On the other hand, when streaked onto another selective media for *S. enterica* (Brilliance™ *Salmonella*), the expected typical morphology and color (purple) was observed. A further qPCR reaction also showed a positive result for *S. enterica*. These two results showed that this was indeed a *S. enterica* strain, albeit atypical, and that this cooked mussel sample was indeed positive for *S. enterica* (Figure 1). The *S. enterica* identity for this sample was further confirmed using an API20E test, showing a typical biochemical profile for *S. enterica*, except for the production of H_2_S, which was also negative in this test. The isolated strain was then serotyped in an accredited laboratory using commercial antisera against somatic and flagellar antigens (Kauffman–White Scheme). Only the antigens of the first flagellar phase were detected, and it was not possible to detect somatic antigens. The final formula obtained with the agglutination test was -:g,s,t:-.

### 3.2. Genome Sequencing and De Novo Assembly

The entire closed genome of SF1060 was obtained by whole-genome sequencing and consisted of five contigs in total (one chromosome and five plasmids) for a total length of 5,018,559 bp. The chromosome size was 4,883,048 bp with a GC% of 52.0, and annotation with Prokka showed 4834 annotated genes, 22 rRNA, 1 tmRNA and 89 tRNA, and 2 CRISPR regions (Table 1).

### 3.3. In Silico Characterization and Antimicrobial Resistance Profiling

In silico serotyping and MLST analyses identified this strain as serotype Senftenberg—somatic and flagellar antigens (1,3,19:g,s,t:-) and belonging to ST14. In silico AMR prediction showed the presence of the *aac(6′)-Iaa*, a cryptic endogenous gene in *S. enterica*. Also, SPI-1, SPI-2, SPI-3, SPI-4, SPI-5, and SPI-9 were detected.

### 3.4. Genetic Basis for Loss of Somatic Antigen Expression

The *rfb* operon is responsible for somatic antigen biosynthesis in *S. enterica* serotype Senftenberg. A pairwise genome alignment with another Senftenberg strain GTA-FD-2016-MI-02533-2 (CP038604) showed a chromosomal re-arrangement that occurred at the *rfb* operon, responsible for O-antigen biosynthesis. The *rfb* operon of strain GTA-FD-2016-MI-02533-2 (CP038604) is composed of genes *rfbB*, *rfbD rfbA*, and *rfbC*. This re-arrangement occurred at the gene *rfbD*, which encodes a dTDP-4-dehydrorhamnose reductase (Uniprot P26392), mediated by an *IS5* family transposase *ISEc68*, resulting in a truncated *rfbD* gene and a separation from the *rfbA* and *rfC* genes, truncating the *rfb* operon (Figure 2).

### 3.5. Analysis of Hydrogen Sulfide Production Pathways

The *phs* operon of strain SF1060, responsible for the reduction of thiosulfate to hydrogen sulfide, did not present premature stops or other obvious disruptive mutations. Comparison with the reference *S. Typhimurium* LT2 sequence (L32188.1) identified several SNPs differences in *phsA* and *phsC* (Figure 3); however, these likely reflect normal sequence divergence between serovars and were not interpreted as causative of the H_2_S-negative phenotype. In the case of *phsA,* there are missense mutations in position 420 (S>A), 421 (A>V) and in the last residue 758 (A>V). For *phsC,* there are missense mutations in position 79 (L>F) and 156 (V>A). The *ars* operon encodes proteins involved in the anaerobic reduction of sulfite, allowing the production of hydrogen sulfide (H_2_S) from sulfite under oxygen-free conditions. In this operon, there are only two missense mutations in position 208 and 219 (H>R in both cases) of gene *arsA*. No nonsense mutations were detected in the *cysJIH* operon either.

### 3.6. Phylogenetic Relationship to Regional and Global S. Senftenberg Strains

**Figure 4:**
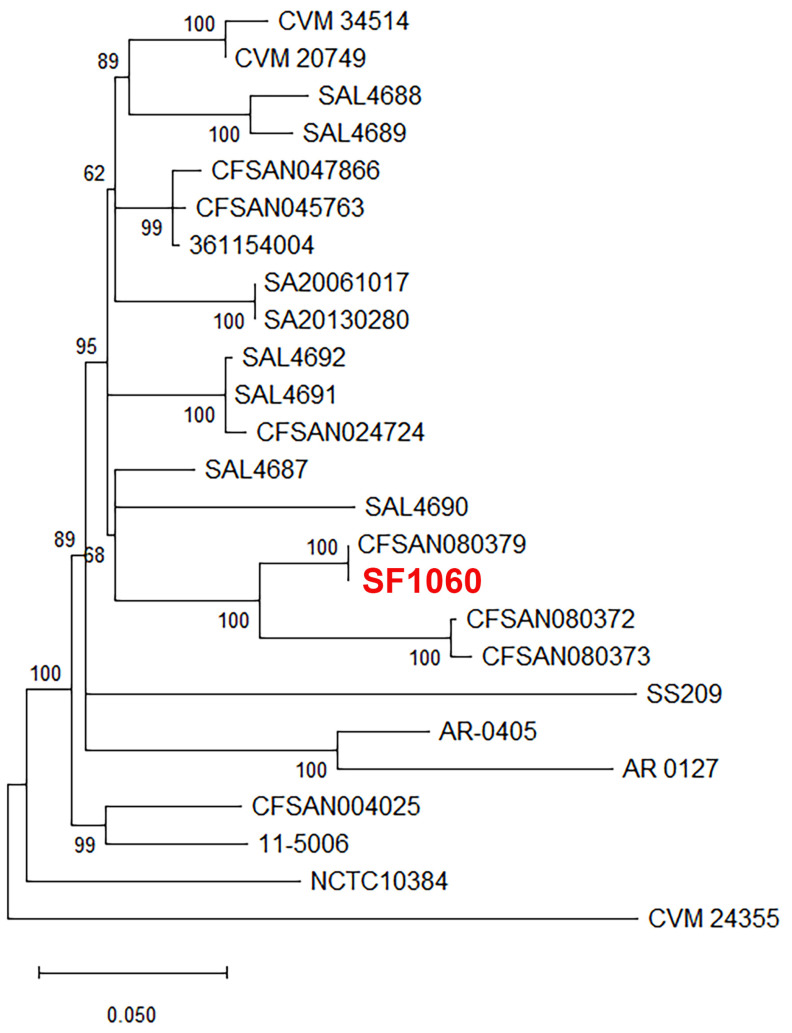
Phylogenetic relationship of *S. enterica* serovar Senftenberg strain SF1060 with global strains based on whole-genome multilocus sequence typing (wgMLST). A total of 3456 core genome coding sequences (CDSs) shared among SF1060 and 24 additional *S.* Senftenberg genomes retrieved from NCBI (Supplementary Table S1) were analyzed. SNPs identified from allelic differences within the shared core genome were used to infer a phylogenetic tree using the Maximum Likelihood method with the Kimura 2-parameter model [18] of nucleotide substitutions and 1000 bootstrap replicates in MEGA12.1 [19]. The analysis included strains from diverse geographic locations (Spain, USA, Canada, Chile, France, United Kingdom) and sources (mussels, clinical samples, avian, food products) spanning 1964–2023. Strain SF1060 (highlighted in red) clustered with strain CFSAN080379, previously isolated from mussels in Galicia, Spain, in 2015, with no SNP differences detected in the shared core genome, suggesting long-term persistence of this ST14 lineage in the regional marine aquaculture environment. wgMLST allele calling was performed using Ridom SeqSphere+ v9.0.8 with pairwise ignoring of missing values. Branch lengths represent genetic distances inferred from core genome SNP variation, and bootstrap support values are shown at the nodes.

A wgMLST analysis showed that SF1060 clustered with another *S.* Senftenberg previously isolated in the region (also with the same chromosomal re-arrangement at the rfb operon) (Figure 4). The phylogenetic analysis included 24 *S.* Senftenberg genomes from diverse sources and geographic locations (Supplementary Table S1), including clinical strains, food samples, and wildlife sources from North America, South America, and Europe. *S.* Senftenberg is commonly isolated from seafood in northwestern Spain, where it accounts for up to 50% of the S. enterica strains identified. Although H_2_S-negative variants have been previously reported, this new strain (SF1060) exhibits additional genetic modifications that complicate not only its detection but also its serotyping.

## 4. Discussion

This study identified and characterized an atypical *S.* Senftenberg strain (SF1060) from cooked mussels that presents dual challenges for conventional detection: loss of somatic antigen expression and absence of H_2_S production. While the genomic basis for the loss of somatic antigen expression is supported by the identified rearrangement within the *rfb* operon, the molecular mechanism underlying the H_2_S-negative phenotype remains unresolved. Although several missense mutations were identified in genes associated with sulfur metabolism, their contribution to the phenotype remains hypothetical and was not functionally validated in this study. Together, these traits illustrate the complexity of *S. enterica* phenotypic variation and the difficulties associated with isolation and identification using conventional phenotypic methods.

The loss of somatic antigen expression in SF1060 can be directly attributed to a chromosomal rearrangement within the *rfb* operon, mediated by an *IS5*-family transposase (*ISEc68*). The *rfb* operon encodes the enzymes involved in O-antigen biosynthesis, and variations within this cluster account for the diversity of somatic antigens observed across the *Salmonella* genus [20]. This single rearrangement event likely disrupted the *rfb* operon through two distinct mechanisms. First, it directly truncated the *rfbD* gene, which would be expected to render its enzyme product non-functional. Second, by separating *rfbA* and *rfbC* from the rest of the operon, it is highly probable that these genes were isolated from their native promoter (Figure 2). Such a separation would be expected to prevent their transcription. The combined effect of a non-functional *rfbD* and the likely loss of expression for *rfbA* and *rfbC* would halt the O-antigen biosynthetic pathway, providing a robust genetic explanation for the serotyping failure. While other studies have reported serologically atypical *S.* Infantis isolated from poultry [21,22], the underlying mechanism was not identified; the rearrangement found here presents a plausible explanation, underscoring the important role mobile genetic elements may play in creating antigenic diversity in *S. enterica* across various environments. While the *IS5*-family transposase-mediated disruption of the *rfb* operon presents a direct and compelling explanation for the serotyping failure, we acknowledge that our analysis did not extend to all genes potentially involved in lipopolysaccharide biosynthesis. However, the observed large-scale rearrangement is the most parsimonious explanation for the untypable phenotype. Likewise, IS-mediated disruption of O-antigen biosynthesis has been documented in *E. coli* O157:H7, where *IS629* insertion caused gene disruption rather than chromosomal rearrangement [23,24].

In contrast, the mechanism for the H_2_S-negative phenotype remains unclear. The reference media commonly used for *S. enterica* isolation is XLD agar, on which colonies typically appear black due to hydrogen sulfide (H_2_S) production from thiosulfate. The H_2_S reacts with iron salts in the medium to form the characteristic black precipitate, facilitating visual identification of presumptive *S. enterica* colonies [5]. In recent decades, multiple strains of H_2_S-negative *S. enterica* serotypes, including *S.* Infantis [25], *S.* Typhimurium [26], and *S.* Senftenberg [27], have been reported. Alterations in the pathways that reduce thiosulfate and sulfite to H_2_S are believed to be responsible for the emergence of these strains. The *phs*, *cysJIH*, and *ars* operons are the main ones involved in this process: the *phs* operon mediates thiosulfate reduction, the *ars* operon reduces sulfite, and *cysJIH* is involved in the final steps of sulfite production [28]. No nonsense mutations or other obvious inactivating mutations were detected in the *phs*, *ars*, or *cysJIH* operons, suggesting that the H_2_S-negative phenotype of SF1060 is not explained by the classical gene-disrupting mechanisms previously reported for atypical *Salmonella* strains. Although several missense differences were observed relative to *S. Typhimurium* LT2, these comparisons involved different serovars and were not used to infer causality. A limitation of our genomic analysis is its focus on the primary operons (*phs*, *ars*, and *cysJIH*) known to be associated with H_2_S production in *Salmonella*. It is conceivable that the identified missense mutations do not result in a functional impact, and that the phenotype is instead caused by mutations in other unexamined genetic loci or disruptions in transcriptional or post-translational regulation. Therefore, the precise mechanism remains to be elucidated, and our findings highlight the need for future functional studies, such as transcriptomics and proteomics, to confirm the role of these mutations and explore other potential causative factors.

The dual atypical phenotype of SF1060 exposes critical vulnerabilities in standard culture-based surveillance, which relies on colony morphology (e.g., black colonies on XLD) and serotyping for identification. Although molecular detection methods for *S. enterica*, primarily based on qPCR, have been developed in recent years, culture-dependent methods remain the gold standard for the detection of this pathogen [4,5]. These methods rely on a combination of non-selective and selective enrichment steps followed by plating on selective and differential agar media. The phenotypic characteristics of SF1060 expose critical vulnerabilities in these standard protocols. The absence of black colony formation on XLD agar could lead to false-negative results if laboratory personnel rely solely on typical colony morphology for presumptive identification. While SF1060 produced characteristic purple colonies on Brilliance™ *Salmonella* agar, demonstrating that chromogenic media can provide complementary detection capabilities, the lack of somatic antigen expression prevented conventional serotyping, which is essential for epidemiological tracking and source attribution. These limitations are not merely academic concerns; they have direct implications for outbreak investigations, where rapid and accurate strain characterization is critical for implementing control measures.

Molecular detection methods offer a robust solution to these challenges. The integration of rapid molecular screening techniques offers an effective strategy to overcome limitations inherent in culture-based diagnostics. Approaches such as quantitative PCR (qPCR) and loop-mediated isothermal amplification (LAMP) have proven particularly valuable in this context [29,30], as they target conserved genetic sequences rather than phenotypic traits. In our study, qPCR successfully identified SF1060 as *S. enterica* despite its atypical characteristics, with a Cq value of 21 from the enriched mussel sample. However, these methods remain constrained by their limited ability to provide comprehensive strain characterization beyond detection. Hence, the implementation of whole-genome sequencing (WGS) workflows, coupled with user-friendly bioinformatics pipelines, presents a promising alternative for the detection, isolation, and detailed analysis of atypical strains [26,31,32,33,34,35]. WGS represents a more comprehensive approach, enabling simultaneous detection, serotyping, antimicrobial resistance profiling, and phylogenetic analysis from a single dataset.

In the case of SF1060, in silico serotyping using SeqSero2 correctly identified the strain as *S.* Senftenberg (I 1,3,19:g,s,t:-), information that was unattainable through conventional methods. Likewise, routine WGS enables the in silico identification of antimicrobial resistance and virulence determinants using databases such as ResFinder [16] and VFDB [36], respectively. The decreasing costs and increasing accessibility of long-read sequencing platforms, such as Oxford Nanopore Technologies, make WGS increasingly feasible for routine implementation in food safety laboratories [37,38,39,40]. A multi-tiered surveillance approach is warranted: (i) molecular screening for rapid detection; (ii) multiple selective media to capture phenotypic variants; (iii) WGS-based characterization; and (iv) periodic protocol re-evaluation. This integrated strategy would enhance surveillance sensitivity and specificity while enabling effective risk management.

The repeated detection of closely related *S.* Senftenberg ST14 strains in the mussel production environment of Galicia, Spain, suggests it is an endemic pathogen adapted to this specific niche. It has been the predominant serotype isolated from the region’s shellfish and processing facilities for over two decades [41,42]. Whole-genome multi-locus sequence typing (wgMLST) analysis supports the possibility of long-term persistence in the mussel-production environment and its ongoing potential to contaminate shellfish, showing that the recently isolated strain SF1060 is closely related to another ST14 strain (CFSAN080379) from 2015, with both sharing an identical chromosomal rearrangement at the *rfb* operon. However, the limited number of closely related isolates available prevents definitive conclusions regarding the endemic persistence of this lineage. The unique selective pressures of the marine environment, such as high salinity, characteristic of mussel production systems, may favor this lineage [43]. Many of these regional strains exhibit a rough morphotype associated with an altered lipopolysaccharide structure, which enhances biofilm formation and environmental survival [41,42]. The presence of other atypical *S. enterica* strains, including some with resistance to last-resort antibiotics such as colistin, has also been reported in this area (7).

Given that Galicia’s mussel aquaculture accounts for over 90% of Spanish production, the presence of this pathogen poses a significant public health and economic risk [6]. *S.* Senftenberg is a notable cause of human salmonellosis in the European Union, with 101 confirmed cases in 2023 [2], and its detection in cooked mussels indicates either post-processing contamination or inadequate thermal treatment. The atypical phenotypes of these persistent strains may lead to systematic underreporting, complicating control efforts. Therefore, enhanced surveillance is essential, requiring an integrated approach that combines targeted monitoring of environmental reservoirs and processing facility biofilms with genomic surveillance to track persistent lineages. Such measures are critical in coastal regions where aquaculture, tourism, and urban development create complex interfaces between human activities and marine ecosystems.

The *IS5*-family transposase-mediated chromosomal rearrangement in SF1060 illustrates how mobile genetic elements generate phenotypic diversity within *S. enterica* populations. Such rearrangements may affect not only serological classification but also host–pathogen interactions and environmental fitness. However, culture-based surveillance methods systematically exclude strains lacking typical phenotypic markers, potentially leading to substantial underreporting of atypical variants. Culture-independent methods, such as shotgun metagenomics applied directly to food samples [40,44,45,46,47,48], could reveal the true extent of this hidden diversity and provide more comprehensive pathogen surveillance.

## 5. Conclusions

The loss of key phenotypic traits, such as the ability to metabolize thiosulfate to hydrogen sulfide and chromosomal rearrangements leading to the absence of somatic antigen expression, present significant challenges for the isolation and characterization of *S. enterica* using conventional phenotypic methods. These limitations underscore the need to integrate whole-genome sequencing into routine surveillance and monitoring programs. The adoption of WGS not only enhances the detection and precise characterization of atypical strains but also strengthens outbreak investigations and contributes to a more comprehensive understanding of *S. enterica* evolution and persistence in the food chain. Such an integrated approach is essential to reinforce surveillance systems, improve outbreak response capacity, and ensure the continued protection of public health. Furthermore, the possible persistence of atypical *S.* Senftenberg variants in marine aquaculture environments highlights the need for One Health surveillance frameworks that integrate genomic data from food, environmental, and clinical sources to effectively track pathogen evolution and transmission across ecological niches.

## Figures and Tables

**Table 1:** Genomic characteristics of *S. enterica* serovar Senftenberg strain SF1060. The complete genome was assembled using hybrid assembly (Oxford nanopore and illumina sequencing) and consists of one chromosome and five plasmids. GC content (%) of each contig.

Contig	Size	^a^ GC%	Genes
1	4,883,048	52.0	4834
2	71,607	53.2	82
3	50,564	53.7	57
4	7539	49.0	10
5	3506	51.3	3
6	2295	50.5	3

^a^ Bacterial genome GC content.

## Data Availability

The complete genome sequence of *S. enterica* serovar Senftenberg strain SF1060 has been deposited in GenBank under BioProject accession number PRJNA1365827, BioSample accession number SAMN53295204. Raw sequencing data are available under SRA accession numbers SRR36084183 (Illumina) and SRR36084182 (Oxford Nanopore Technologies). The assembled genome is available under accession number ACBLBR000000000. All data supporting the findings of this study are available within the article and its Supplementary Materials.

## Supplementary Materials

The following supporting information can be downloaded at: https://www.mdpi.com/article/10.3390/microorganisms14061284/s1, Table S1: Salmonella enterica serovar Senftenberg genomes used for phylogenetic analysis. Figure S1: Phylogenetic relationship of *S. enterica* serovar Senftenberg strain SF1060 with global strains based on whole genome multilocus sequence typing (wgMLST).
