# Supplementary material for: Genomic Characterization of an O-Antigen-Deficient, Hydrogen Sulfide-Negative Salmonella enterica Serovar Senftenberg Isolated from Cooked Mussels
Source: Microorganisms. 2026 Jun 6;14(6):1284. doi: 10.3390/microorganisms14061284 (PMC13304406; doi:10.3390/microorganisms14061284)
Supplement: Supplementary file 1 [file microorganisms-14-01284-s001.zip › microorganisms-4275846-supplementary.pdf]

Supplementary material

Supplementary Table S1. Salmonella enterica serovar Senftenberg genomes used for phylogenetic analysis. Genomes were retrieved from NCBI and represent diverse geographic locations, isolation sources, and time periods (1964-2023). All genomes were analyzed using whole genome multilocus sequence typing (wgMLST) to assess phylogenetic relationships with strain SF1060.

Sample	Date of isolation	Source	Country	Biosample Number
11-5006	2016	clinical	Canada	SAMN06030139
361154004	2009	pistachio	USA	SAMN01816110
AR_0127	2018	unknown	USA	SAMN04014968
AR-0405	2019	unknown	USA	SAMN12648955
CFSAN004025	2003	fish meal	Mexico	SAMN02352702
CFSAN024724	2012	Kelp Gull	Chile	SAMN03079669
CFSAN045763	2016	raw pistachio	USA	SAMN04451263
CFSAN047866	2016	pistachio	USA	SAMN04549535
CFSAN080372	2015	mussels	Spain	SAMN10875712
CFSAN080373	2015	mussels	Spain	SAMN10875713
CFSAN080379	2015	mussels	Spain	SAMN10875716
CVM 20749	2002	turkey	USA	SAMN14504550
CVM 24355	2002	turkey	USA	SAMN14504707
CVM 34514	2004	turkey	USA	SAMN14504879

NCTC10384	1964	feces	United Kingdom	SAMEA2517359
SA20061017	2006	Avian	Canada	SAMN11029488
SA20130280	2012	Avian	Canada	SAMN11029489
SAL4687	2010	Kelp gull	Chile	SAMN03079663
SAL4688	2011	Kelp Gull	Chile	SAMN03079664
SAL4689	2012	Kelp gull	Chile	SAMN03079665
SAL4690	2009	Homo sapiens	Chile	SAMN03079666
SAL4691	2009	clinical	Chile	SAMN03079667
SAL4692	2012	Kelp gull	Chile	SAMN03079668
SF1060	2023	cooked mussels	Spain	SAMN53295204
SS209	2012	unknown	France	SAMEA2272617

Supplementary Figure S1. Phylogenetic relationship of *S. enterica* serovar Senftenberg strain SF1060 with global strains based on whole genome multilocus sequence typing (wgMLST). Neighbor-joining (NJ) tree constructed using Nei's DNA distance method based on allelic differences across 3,456 core genome coding sequences (CDSs) shared among SF1060 and 24 additional *S.* Senftenberg genomes retrieved from NCBI (Supplementary Table 1). The analysis included strains from diverse geographic locations (Spain, USA, Canada, Chile, France, United Kingdom) and sources (mussels, clinical samples, avian, food products) spanning 1964-2023. Strain SF1060 (highlighted in red) clustered with strain CFSAN080379 with no allelic differences detected by wgMLST, which was isolated from mussels in the same geographic region (Galicia, Spain) in 2015, indicating long-term persistence of this ST14 lineage in the regional marine aquaculture environment. The tree was constructed using Ridom SeqSphere+ v9.0.8 with pairwise ignoring of missing values. Branch lengths represent genetic distances based on the number of allelic differences in core genes.

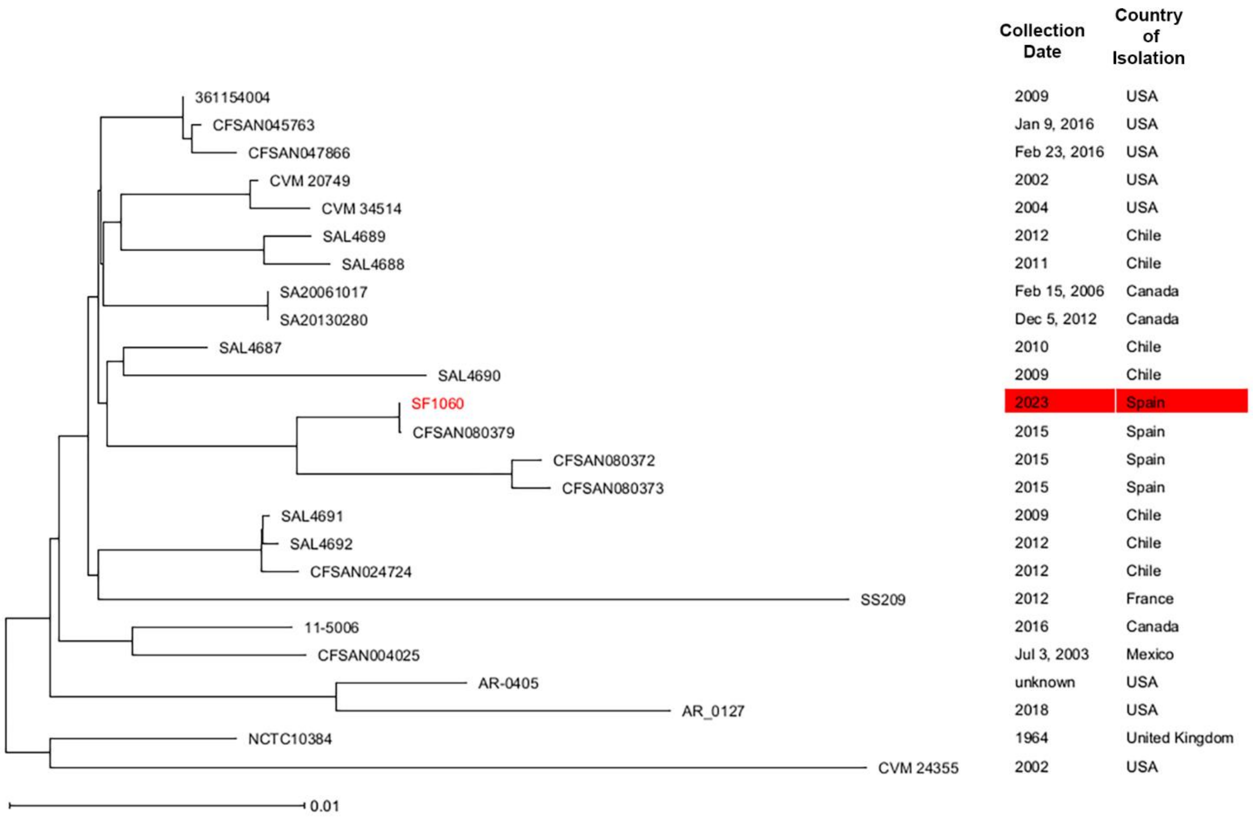
Collection Date
Country of Isolation
361154004
2009
USA
CFSAN045763
Jan 9, 2016
USA
CFSAN047866
Feb 23, 2016
USA
CVM 20749
2002
USA
CVM 34514
2004
USA
SAL4689
2012
Chile
SAL4688
2011
Chile
SA20061017
Feb 15, 2006
Canada
SA20130280
Dec 5, 2012
Canada
SAL4687
2010
Chile
SAL4690
2009
Chile
SF1060
2023
Spain
CFSAN080379
2015
Spain
CFSAN080372
2015
Spain
CFSAN080373
2015
Spain
SAL4691
2009
Chile
SAL4692
2012
Chile
CFSAN024724
2012
Chile
SS209
2012
France
11-5006
2016
Canada
CFSAN004025
Jul 3, 2003
Mexico
AR-0405
unknown
USA
AR_0127
2018
USA
NCTC10384
1964
United Kingdom
CVM 24355
2002
USA
0.01
